# Supplementary material for: The SR Splicing Factors: Providing Perspectives on Their Evolution, Expression, Alternative Splicing, and Function in Populus trichocarpa
Source: Int J Mol Sci. 2021 Oct 21;22(21):11369. doi: 10.3390/ijms222111369 (PMC8583155; doi:10.3390/ijms222111369)
Supplement: Supplementary file 1 [file ijms-22-11369-s001.zip › Supplemental Figure S1 .pdf]

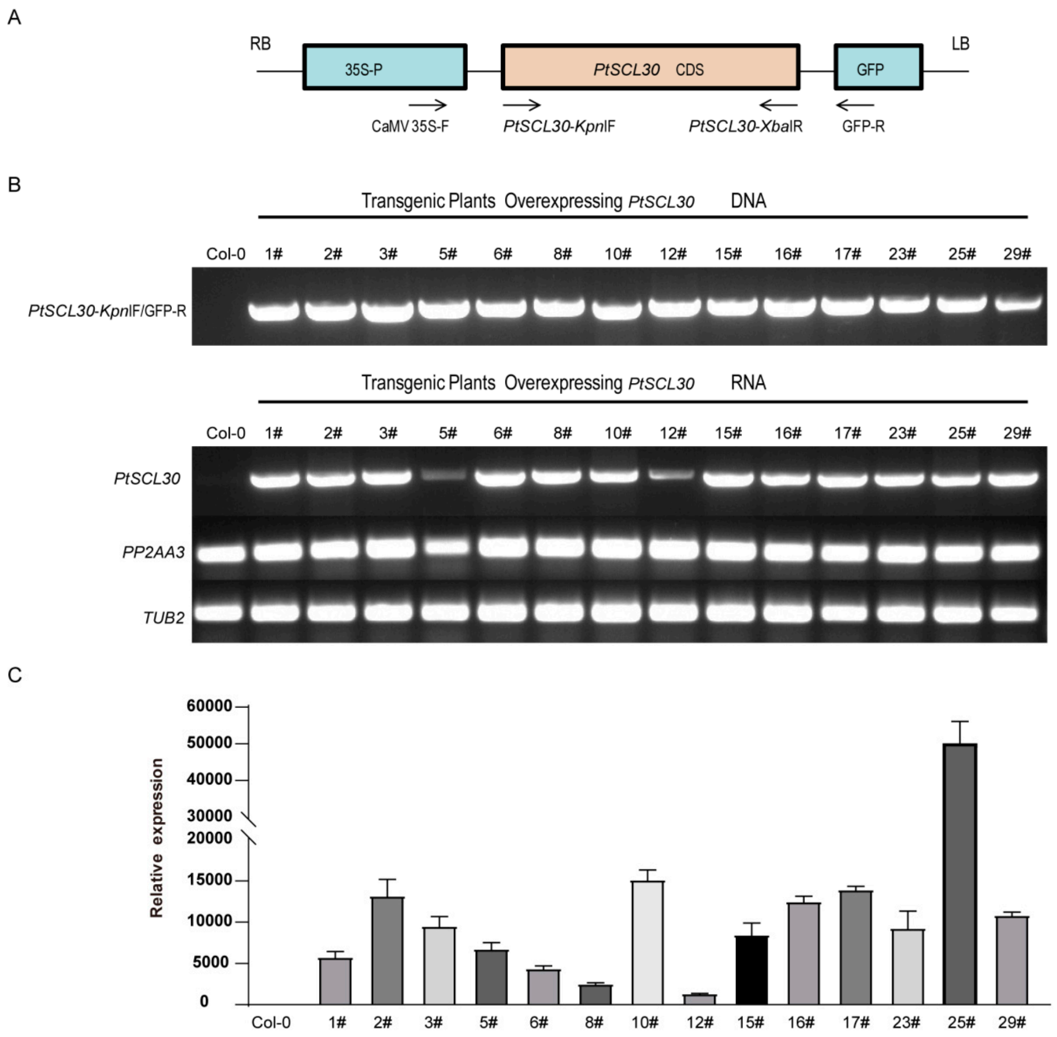

Figure S1. Construction of *PtSCL30* overexpression vector and identification of transgenic plants in *Arabidopsis.* (**A**) Schematic diagram of *PtSCL30* overexpression vector. The *PtSCL30* CDS was amplified by PCR with the following primers: *PtSCL30-Kpn*IF and *PtSCL30-Xba*IR. The *PtSCL30* CDS region was cloned in the *Kpn*I and *Xba*I sites of the pCAMBIA1300-sGFP vector. The arrows indicate forward and reverse primers (CaMV35S-F, *PtSCL30-Kpn*IF, *PtSCL30-Xba*IR and GFP-R) which were used in PCR analysis. 35S-P: the 35S cauliflower mosaic virus promoter; RB: right border; LB: left border; GFP: green fluorescent protein. (**B**) Genomic DNA and RNA PCR analysis of Col-0 and 35S::*PtSCL30* OE lines. The genomic DNA and total RNA were isolated from rosette leaves and used in PCR analysis with the primer pairs: *PtSCL30-Kpn*IF with GFP-R and *PtSCL30-Kpn*IF with *PtSCL30-Xba*IR, respectively. (**C**) Relative expression levels of the *PtSCL30* gene in transgenic *Arabidopsis*. Error bars represent the standard deviations of three biological replicates. The genes *PP2AA3* (*AT1G13320*) and *TUB2* (*AT5G62690*) were used to measure the quality and quantity of cDNA and served as reference genes for normalization of the expression. The numbers represented the different lines of transgenic *Arabidopsis*.
